# From Information Exposure to Protective Behaviors: Investigating the Underlying Mechanism in COVID-19 Outbreak Using Social Amplification Theory and Extended Parallel Process Model

**DOI:** 10.3389/fpsyg.2021.631116

**Published:** 2021-05-25

**Authors:** Shuguang Zhao, Xuan Wu

**Affiliations:** ^1^School of Journalism and Communication, Nanjing University, Nanjing, China; ^2^Zijin Media Research Institute, Nanjing University, Nanjing, China

**Keywords:** COVID-19, social amplification theory, extended parallel process model, protective behavior, news media, personal network

## Abstract

Ever since the outbreak of the coronavirus disease (COVID-19), people have been flooded with vast amounts of information related to the virus and its social consequences. This paper draws on social amplification theory and the extended parallel process model (EPPM) and assesses the following: (a) how two amplification stations—news media and peoples’ personal networks—influence the risk-related perceptions of people (perceived efficacy and perceived threat) and (b) how these risk-related perceptions impact people’s health-protective behaviors. This study surveyed 1,946 participants. The results indicate that peoples’ exposure to news media significantly and positively predicted both perceived efficacy and perceived threat. It also shows that peoples’ exposure to risk-related information through their personal networks negatively predicted their perceived efficacy, but it positively predicted their perceived threat. The mediating effect of fear was examined, and the result was contradictory to the EPPM. In short, this study reveals the underlying mechanism of individuals’ exposure to risk information, processing, and precautionary measures.

## Introduction

Ever since the human-to-human transmission of coronavirus disease (COVID-19) was officially confirmed and announced at the end of January 2020 (China Daily, 2020), the spread of COVID-19 and its social consequences have become the central topic of media coverage worldwide. Coverage has been so intense and persistent that people are all exposed to it through different channels such as news media and personal networks, either intentionally or passively. The social amplification theory (SAT) suggests that peoples’ perceptions of risk can be amplified or weakened depending on the channels through which they receive risk-related information (Renn et al., 1992). Furthermore, according to the extended parallel process model (EPPM), people’s perceived threat regarding a risk and the efficacy of countermeasures determine their responses to risks (Witte, 1992). Moreover, fear plays an important role in this process (Witte, 1994). These two theories together can map the route between peoples’ exposure to risk information and their health-protective behaviors and thus provide a useful framework in understanding people’s information consumption and responses to the outbreak of the COVID-19 pandemic in China.

Although the media has been accused of overamplifying panic, fear, and anxiety during the spread of this pandemic (Blagojević, 2020; Richtel, 2020), the SAT suggests that peoples’ personal networks also have an amplifying effect on their perception relating to the pandemic. However, while many studies on risk communication have investigated how media influenced individuals’ risk perceptions and subsequent coping behaviors (e.g., He et al., 2020; Huang, 2020), there is still lack of discussions on another key channel of personal network. As COVID-19 is a new yet most serious challenge for global public health, the findings in the existing literature are not necessarily applicable to the present unprecedented situation of a global pandemic. This paper seeks to fill these gaps by focusing on how peoples’ exposure to information through different channels influences their perceived threat and efficacy regarding COVID-19, how their threat and efficacy perceptions of COVID-19 influence the affective factor (in this case, fear), and fear’s mediating role between peoples’ perceptions of COVID-19 and their protective behaviors. This study is significant in several ways. First, it reveals news media and people’s personal networks’ effects on how people cognitively process, affectively react, and behave in the context of a pandemic, providing a timely and valuable insight. Second, by adopting and integrating concepts from both the SAT and EPPM, this study introduces an integrated and extensive framework for studies of risk-related information, perception, and behaviors in public health emergencies, thereby enriching the literature on health communication. The lack of research on the mechanisms underlying how peoples’ exposure to information and processing of that information affect their adoption of precautionary measures has been highlighted in previous research (e.g., Zhang et al., 2015; Yoo et al., 2016; Iorfa et al., 2020; Kim et al., 2020), and the current study aims to fill the gap.

## Literature Review

### Effects of Amplification Stations on Risk-Related Perceptions

#### Social Amplification Theory

The social amplification theory—also known as the social amplification risk framework—aims to explore why the public intensifies some risks and ignores others. SAT denotes that “information processes, institutional structures, social group behavior, and individual responses shape the social experience of risk, thereby contributing to risk consequences” (Renn, 1991, p. 289). In other words, risk experience not only is about physical harm but also subjects to personal interpretation of risks (Kasperson et al., 1988). The SAT further suggests that the signals, as a message, are sent through transmitters and that these transmitters alter the original message by intensifying or attenuating some signals before passing them on (Kasperson et al., 1988). During this process, certain aspects of messages are highlighted, reinterpreted, or elaborated, and these alterations influence the message’s receivers’ perceptions of risk (Kasperson et al., 2003).

SAT differs between individual and social amplification stations. The former follows their personal values and interpretive patterns, while the latter perceives risk-related information according to the rules of the social groups and institutions of which they are a part and their roles within these groups and institutions (Renn et al., 1992). Examples of amplification stations include scientists, risk management institutions, the news media, activists and social justice organizations, opinion leaders within social groups, personal networks, and public agencies (Kasperson et al., 1988). The SAT’s influence in risk communication research has been well documented (Raupp, 2014); therefore, it is an appropriate basis for our study.

#### How the News Media and Peoples’ Personal Networks Function as Amplification Stations

As people learn about the risks and risk events mainly from various media outlets rather than direct experience, mass media, particularly news media, as an amplification station is frequently investigated in previous communication studies (Kasperson and Kasperson, 1996). Binder et al. (2014) reviewed the literature on the news media as amplification stations and found that the literature mainly focused on the following: (a) the amplification or attenuation of risk, (b) comparing the objective risk events with the volume and tone of media coverage, and (c) news media’s effects on individuals’ risk perceptions. They found that the third topic has been heavily investigated in recent years. News media was found to influence the public’s risk perception through the sheer amount of news reported, the contents, and the tone of these reports (Reintjes et al., 2016). Some researchers have investigated the impact of different types of media on individuals’ risk perceptions; for example, Snyder and Rouse (1995) suggested that entertainment media tended to influence personal-level risk perceptions and the news media tended to influence societal-level risk perceptions. Oh et al. (2015) likewise found that entertainment media indirectly influenced peoples’ personal-level risk perceptions through the emotional dimensions of risk characteristics in the case of H1N1 coverage in South Korea. Morton and Duck (2001) found that exposure to information through newspapers strongly predicted peoples’ personal perceptions of the risk of skin cancer, and Coleman (1993) found that television news was a more powerful predictor of peoples’ risk perception than peoples’ interpersonal communication.

However, there are still several research gaps in the existing literature. First, news media in previous studies often referred to newspaper and television, but nowadays there are news outlets that are entirely based on social media. Moreover, traditional media agencies also have their online presences, which are less specified in previous studies. Second, the topics covered by health communication researchers vary, and many focused on non-urgent risks rather than global public health emergencies like the COVID-19 pandemic (Li, 2018). Third, despite the fact that peoples’ personal networks are important information sources, when there is a lack of direct experiences of risks (Kasperson et al., 1988; Young et al., 2008), it is found that only limited empirical research has been conducted on how communication about a pandemic through personal networks influences people’s perceptions of risk. To this end, this study investigates both news media and personal network’s influence on two dimensions of people’s risk-related perceptions—perceived threat and perceived efficacy—during the COVID-19 pandemic in China. The research questions were proposed as follows:

RQ1: How does people’s exposure to COVID-related information through news media predict their (a) perceived efficacy and (b) perceived threat?

RQ2: How does people’s exposure to COVID-related information through their personal networks predict their (a) perceived efficacy and (b) perceived threat?

### The Path From Risk Perceptions to Health-Protective Behaviors

#### The Extended Parallel Process Model

The EPPM is a prominent theory in health communication research (Rintamaki and Yang, 2014), which was proposed by Witte in 1992. It provides a framework that helps to understand why fear, as an emotion, fails to or succeed in changing people’s risk-related behaviors (Popova, 2012). It consists of several constructs, including fear, perceived threat (e.g., the perceived severity of a risk and self-perceived susceptibility to a risk), perceived efficacy (self-efficacy and response efficacy in general), and two types of responses (fear control and danger control) (Witte, 1992). Specifically, EPPM posits that if the risk event is considered as an irrelevant or trivial threat, people will take no responses; otherwise, they tend to evaluate their efficacy regarding the risk, which leads to different responses. Especially when the perceived efficacy is greater than the threat, people will take control of the perceived danger, which will result in their protective behavior regarding the risk, whereas if it is the other way around, people tend to control their fear, which may lead to avoidance of the risk messages and denial of protective behaviors (Witte, 1994). The EPPM offers a theoretical framework for predicting individuals’ responses based on the perceptions of risks (Maloney et al., 2011) and has been broadly applied in a variety of health contexts (Rintamaki and Yang, 2014). These include cancer risks (Kline and Mattson, 2000; Morman, 2000; Hubbell, 2006), HIV/AIDS (Cho and Witte, 2005; Smith et al., 2007), and influenza vaccination (LaVela et al., 2007; Cameron et al., 2009).

#### Perceptions of Threat, Efficacy, and Health Protective Behaviors: The Moderating Role of Fear

Peoples’ perceptions of risks have been found to be reliable predictors of their preventive behaviors in prior studies on public health emergencies (Rimal, 2001). For example, Witte et al. (2002) investigated preventative behaviors from HIV/AIDS in Ethiopia and found that perceived susceptibility, self-efficacy, and response efficacy were significant predictors of condom use among Ethiopian urban youth. Similarly, Bults et al. (2011) found that people with higher levels of self-efficacy tended to take more preventive measures in the context of the H1N1 flu in the Netherlands. Hubbell (2006) showed that Mexican-American women were less likely to avoid seeking cancer-related information when they had high perceptions of the severity of breast cancer and their susceptibility to it, and Li (2018) found that perceived self-efficacy and proxy efficacy were positive predictors of peoples’ danger control outcomes and that the latter negatively predicted peoples’ fear control outcomes in the context of Asian responses to Ebola outbreaks.

**FIGURE 1:**
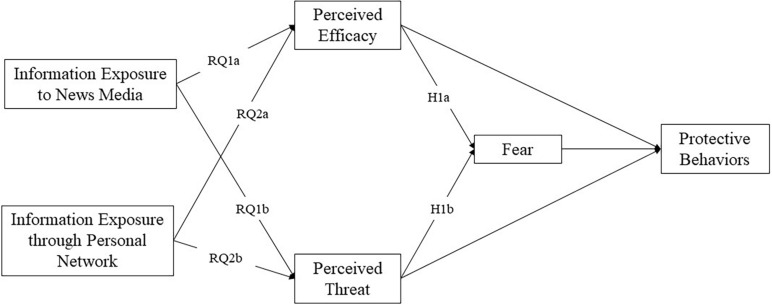
Hypothesized path model, research questions, and hypotheses. H2a and H2b tested mediation effects, therefore were not directly reflected in the path model.

Unlike its predecessor—protection motivation theory, which posits that fear plays a minimal and indirect role—the EPPM gives fear more weight (Witte, 1992; Lewis et al., 2007). Previous studies have investigated fear’s potential effects on people’s health-related behavior in different settings. For instance, Witte (1994) found that fear did not influence people’s message acceptance outcomes regarding HIV/AIDS but that fear had indirect and significant impacts on high-efficacy participants. Zhang et al. (2015) found that frequent exposure to media coverage during the H1N1 flu outbreak evoked more fear, which subsequently led to the implementation of preventive actions (Zhang et al., 2015). Understanding about the mediating effects of fear on health behaviors can reveal a complete picture of how individuals’ cognition affects their health behaviors. More importantly, such examination in the Asian context during the global pandemic outbreak not only enriches the literature of public health emergencies but also provides timely insights for policymakers and practitioners. To this end, this research proposed the following hypotheses, and the complete model of this research can be found in Figure 1.

H1a: Perceived efficacy is negatively associated with fear.

H1b: Perceived threat is positively associated with fear.

H2a: Fear mediates between perceived efficacy and protective behavior.

H2b: Fear mediates between perceived threat and protective behavior.

## Materials and Methods

### Sampling

This study employed quota sampling. The sample was designed based on the populations of each province and/or municipality in Mainland China according to the National Bureau of Statistics. Survey data were collected online *via* a well-known Chinese survey firm, Sojump, between February 25 and 28, 2020. The minimum age of the participants was 18 years. Approval from the Institutional Review Board was obtained before collecting data for the survey. After excluding invalid cases (e.g., incomplete questionnaires), the data of 1,946 participants were used for further analysis. In all, 63% of participants were male, over 90% had ages between 18 and 40 years, and 38.5% had an educational qualification of bachelor’s degree.

### Measures

#### Risk Information Exposure

To measure people’s exposure to risk-related information through both news media and their personal networks, the participants were asked about how frequently they had been exposed to COVID-related information in the past month through different sources, including print media (e.g., newspaper, magazines), broadcasts, cable TV, news apps/websites, Weibo/Wechat account of news media, spouses or boy/girlfriend, parents/siblings/children, best friends, relatives, and other acquaintances. Some of our survey items were adopted from Wu and Li (2017), who examined the effects of mass media and social media exposure on risk perception toward the haze issue in China. A seven-point Likert scale was used, with 1 being “never” and 7 being “always.” The reliabilities (Cronbach’s α) for news media (*M* = 4.63, *SD* = 1.21) and personal network (*M* = 4.41, *SD* = 1.33) scales were 0.73 and 0.88, respectively.

#### Perceived Efficacy

The variable of perceived efficacy has two dimensions: perceived self-efficacy and perceived response efficacy. Each dimension was measured by three items adopted from Witte (1996b), with some wording changes to fit the context of COVID-19. The participants responded to these items *via* a seven-point Likert scale (where 1 = “strongly disagree” and 7 = “strongly agree”). The scores of the two dimensions were averaged and combined into a single score for perceived efficacy: the higher the combined score, the higher a participant’s level of perceived efficacy. The reliabilities for the subscales of perceived self-efficacy (*M* = 5.72, *SD* = 1.23) and perceived response efficacy (*M* = 5.71, *SD* = 1.24) were 0.87 and 0.88, respectively. The full scale of perceived efficacy (*M* = 5.72, *SD* = 1.19) showed a high Cronbach’s α value at 0.93.

#### Perceived Threat

The scale for measuring perceived threat was also adopted from Witte (1996b), with a few changes in the wording of the manuscript. The perceived threat consists of two dimensions: perceived susceptibility and perceived severity of the disease, and three items were used for each dimension. A seven-point Likert scale was used, with 1 being “strongly disagree” and 7 being “strongly agree.” Each dimension of perceived threat was averaged and combined into a single score: the higher the combined score, the higher a participant’s level of perceived threat. The reliabilities for the subscales of perceived susceptibility (*M* = 3.21, *SD* = 1.60) and perceived severity of COVID-19 (*M* = 5.06, *SD* = 1.47) were 0.87 and 0.88, respectively. The full scale of perceived threat (*M* = 4.13, *SD* = 1.13) showed a Cronbach’s α value of 0.74.

#### Fear

Fear was measured by the items adopted from McMahan et al. (1998). The participants were asked how “frightened,” “scared,” and “anxious” they were about COVID-19. They responded using a seven-point Likert scale (1 = “strongly disagree” and 7 = “strongly agree”). The mean and standard deviation for this variable were 4.13 and 1.46, respectively, and the Cronbach’s α value was 0.91.

#### Protective Behavior

We measured health-protective behavior with the activities promoted by the World Health Organization (World Health Organization, 2019), including handwashing, wearing a face mask, covering the mouth and nose when sneezing, and social distancing. The participants were asked about how frequently they had employed these behaviors in the past month *via* a seven-point Likert scale (1 = “never” and 7 = “always”). The mean and standard deviation for this variable were 5.94 and 1.19, respectively, and the Cronbach’s α value was high at 0.91.

#### Control Variables

Age, gender, and education were used as control variables in this study. Age was measured using a seven-point Likert scale (1 = “18–23” and 7 = “61 and above”), and education was measured in a similar way (1 = “primary school and below” and 7 = “postgraduate”).

### Data Analysis

To examine hypothesized relationships in the model, a path analysis was conducted using AMOS 23.0 software. As most of the variables were measured by established scales, the mean values of the variables were used as observed variables instead of latent constructs in the analysis. Prior to the path analysis, confirmatory factor analysis (CFA) was conducted to validate the measurement model, with six latent variables constructed by 29 observed indicators. The criterion of Fornell and Larcker, 1981 was used to assess the convergent validity based on three factors: factor loadings (standardized regression weights), reliability (Cronbach’s alpha), and average variance extracted (AVE); the discriminant validity was assessed by comparing values of AVE and maximum shared squared variance (MSV), and the former should be larger than the latter to indicate good discriminant validity. The results showed that all factor loadings were above 0.7 (except for one factor loading that was 0.530 but which is acceptable since it passed the threshold of 0.5), AVE values were above 0.7, and values of Cronbach’s alpha were above 0.7, thereby indicating a good convergent validity. The comparison between AVE and MSV showed that, in general, AVE values are larger than MSV values; as the AVE value of perceived behavior (PB) was quite close to MSV between PB and perceived efficacy (PE), it is also considered as acceptable. The construct of information exposure to news media had a slightly smaller AVE value than one of its MSV values. While deleting the one indicator (information exposure to news apps/websites) with a lower factor loading may improve the result, however, considering that news apps/websites are an important and frequently used information channel, this indicator was retained in this research. Given that chi-square test tends to over-reject large samples (Bentler and Bonett, 1980), the following criteria were used to evaluate the model fit: root mean square error of approximation (RMSEA), standardized root mean square residual (SRMR), and comparative fit indexes (CFI) (Hu and Bentler, 1999). According to Kline (2015), a RMSEA value of 0.05 or less indicated a close approximate fit, and values between 0.05 and 0.08 suggested a reasonable error of approximation. Hu and Bentler (1999) pointed out that “a cut of value close to 0.95 for CFI…a cutoff value close to 0.08 for SRMR” (p. 27) were considered as indicators for an adequate model fit. The results of the CFA showed an overall acceptable measurement model: CFI = 0.965, RMSEA = 0.046 (90% CI: 0.044–0.048), SRMR = 0.085. After the validation of measurement model, the complete structural model was tested, and the path model showed an acceptable fit: CFI = 0.961, RMSEA = 0.076 (90% CI: 0.066—0.086) SRMR = 0.047.

## Results

### Effects of Demographic Variables

The demographic variables were included in the path model as control variables, and their relationships with PE, perceived threat (PT), fear, and PB were also examined. The results indicated that age was significantly and positively related to PT (β = 0.076, *p < 0*.001, *SE* = 0.024, 90% CI: 0.036–0.114) and that education level was positively related to both PE (β = 0.127, *p < 0.*001, *SE* = 0.023, 90% CI: 0.088–0.164) and PT (β = 0.080, *p < 0*.001, *SE* = 0.022, 90% CI: 0.043–0.116) but negatively associated with fear (β = −0.054, *p < 0*.01, *SE* = 0.020, 90% CI: 0.088–0.020).

### Research Questions and Hypotheses

RQ1a and RQ1b focused on how peoples’ exposure to news media about COVID-19 influenced their PE and PT. The results of the path analysis showed that exposure to news media is a significant predictor of both PE (β = 0.125, *p < 0*.001, *SE* = 0.031, 90% CI: 0.078–0.182) and PT (β = 0.100, *p < 0*.001, *SE* = 0.036, 90% CI: 0.040–0.159): the more the exposure to news media, the higher the participants’ levels of PE and PT.

RQ2a and RQ2b aimed to understand how information exposure through personal network predicted PE and PT. The results showed that information exposure through personal network was a positive predictor of PT (β = 0.104, *p < 0*.01, *SE* = 0.036, 90% CI: 0.043–0.162) but a negative predictor of PE (β = −0.249, *p < 0*.001, *SE* = 0.031, 90% CI: −0.302 to −0.199). In other words, more exposure to information on COVID-19 *via* personal network leads to less PE but more PT.

**FIGURE 2:**
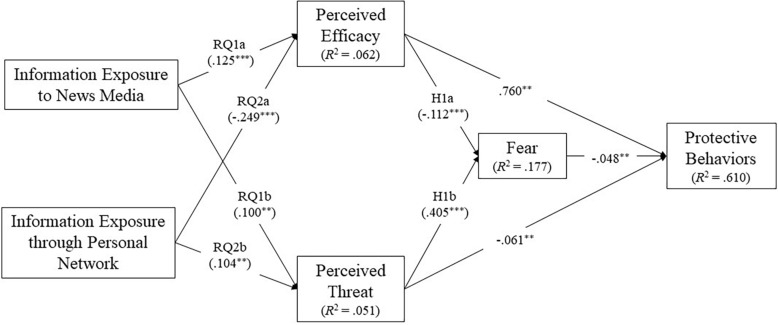
Path model estimation results. Significance: *p < 0.05, **p < 0.01, ***p < 0.001. H2a and H2b tested mediation effects, therefore were not directly reflected in the path model. The detail results of H2a H2b can be found in Table 1.

H1a and H1b proposed a negative association between PE and fear as well as a positive relationship between PT and fear. The analysis supported both hypotheses: PE was found to be a significant negative predictor of fear (β = −0.112, *p < 0*.001, *SE* = 0.020, 90% CI: −0.145 to −0.079), and PT was found to be a significant positive predictor of fear (β = 0.405, *p* < 0.001, *SE* = 0.022, 90% CI: 0.368–0.440). H2a and H2b focused on the mediating role of fear. To investigate this mediating effect, a bootstrap test was conducted using 2,000 bootstrap resamples. Fear was found to be a negative predictor of PB (β = −0.048, *p* < 0.01, *SE* = 0.013, 90% CI: −0.071 to −0.027). The results also indicated significant indirect effects from PE to PB *via* fear (β = 0.005, *p* < 0.01, *SE* = 0.002, 90% CI: 0.003–0.009) and from PT to PB *via* fear (β = −0.019, *p < 0*.01, *SE* = 0.005, 90% CI: −0.029 to −0.011) as well as significant direct effects from PE to PB (β = 0.760, *p < 0*.01, *SE* = 0.013, 90% CI: 0.737–0.781) and from PT to PB (β = −0.061, *p < 0*.01, *SE* = 0.017, 90% CI: −0.088 to −0.035). The details of the direct and indirect effects of PE and PT on PB can be found in Table 1. Statistically, the indirect effect from both PE to PB and PT to PB was significant. To further understand the mediation effect of fear, the effect sizes of the mediator were calculated using the P*_*M*_* criterion (the ratio of indirect effect to the total effect) as proposed by Wen and Fan (2015). The results indicated that the effect size of fear from PE to PB was 0.65% and that from PT to PB was 23.75%. Obviously, fear explained a minimum part of the total effect in the relationship from PE to PB; it is also notable that despite the effect size of 23.75%, fear is hardly regarded as a mediator due to the overall small total effect of the relationship from PT to PB. Considering the results above, it is reasonable to conclude that fear should not be identified as a mediator despite the significant indirect effect in the relationship of PE to PB and PT to PB, which rejected H2a and H2b. Details of hypotheses testing results can be found in Figure 2.

**TABLE 1:** Standardized direct and indirect effects of perceived efficacy (PE) and perceived threat (PT) on perceived behavior (PB), with fear as the mediator.

	***p-*value**	**Estimate**	**SE**	**CI**
**Effects from PE to PB**				
Total effect	0.002	0.766	0.013	0.743–0.786
Direct effect	0.001	0.760	0.013	0.737–0.781
Indirect effect	0.001	0.005	0.002	0.003–0.009
**Effects from PT to PB**				
Total effect	0.001	−0.080	0.015	−0.105 to −0.056
Direct effect	0.001	−0.061	0.017	−0.088 to −0.035
Indirect effect	0.001	−0.019	0.005	−0.029 to −0.011

**Estimate, point estimate; lower, lower boundary of the 90% confidence interval; upper, upper boundary of the 90% confidence interval. CIs are bias-corrected 90% confidence intervals based on 2,000 bootstrap samples. N = 1,946.**

## Discussion

This paper aimed to answer two questions. First, how do different amplification stations influence people perceptions of health-related risks? Second, how do these perceptions impact people’s protective behaviors? By focusing on these two questions, this research examined the path from information receiving to behavior change in people, thereby contributing to the understanding of their responses to health risks.

This study focused on the effects of two different amplification stations, namely, news media and personal network, on individuals’ PE and PT. The results indicated that the increase in exposure to news media led to a higher level of both perceived efficacy and threat, whereas the increase in exposure to information through personal networks lowered people’s efficacy and increased threat perceptions. These results highlighted the role of personal network in influencing individuals’ risk perceptions. Specifically, the media is often criticized for creating unnecessary panic among the public (Zhang et al., 2015); however, the findings of this study indicated that people’s exposure to COVID-related information through their personal networks may be more likely to induce unpleasant cognitive reactions. There are some possible explanations for these findings. First, due to the media censorship in China, it is not uncommon for people to believe that information about public events (such as COVID) available in official news media is carefully selected and worded so as to avoid inducing panic and chaos. In this case, personal network is often a preferred channel to obtain the information that may be “covered” or “filtered,” and people usually have the tendency to accept the information from personal network more easily. If the information is exaggerated, biased, or even fabricated (which is true in many cases), it is more likely to induce negative emotions. Second, the characteristics of language used in the context of personal network could be another possible reason for these findings. The communication within personal network is often informal and could be vivid, personalistic, and specific with intense language, which has great potential for strong fear appeals (Witte, 1996a). This kind of information is more “attention getting, emotionally arousing, interesting, image producing, (and) memorable” (Keller and Block, 1997, p. 296) and therefore could be more persuasive when people form their perceptions regarding the pandemic. Lastly, psychological distance may be another key factor for explaining these results. People usually perceive less psychological distance with those who are socially proximal, and interpersonal discussions about the risks often involve personal stories and experiences; therefore, the information from such channel will be deemed as more self-relevant and trustworthy, which increased the likelihood of undesirable cognitive reactions (Cho et al., 2013). The findings of this research highlighted the importance of personal network as an amplification station, and its role in the development of people’s risk perceptions is worth more attention.

This study also investigated the relationships among people’s PE, PT, fear, and PB. The results indicated that perceived efficacy was negatively related to fear, whereas the perceived threat showed a positive association with fear, which are consistent with the propositions of the EPPM. Moreover, it was noted that, as fear increased, the protective behavior decreased. According to the EPPM, the fear appeal leads to both danger control and fear control, and the meta-analysis of previous research on fear appeals by Witte and Allen (2000) suggested that, as fear increased, it produced stronger fear control than danger control. In other words, when increasing fear makes people believe that it is futile to control the danger, they tend to focus on eliminating their fear through denial, defensive avoidance, or reactance (Witte and Allen, 2000), therefore leading to decreased protective behaviors. Moreover, this research also found a positive association between PE and PB, and it supported the results in previous research that “the weaker efficacy message, the greater the fear control/defensive responses” (Witte and Allen, 2000, p. 598).

Additionally, the mediation effect of fear was also examined in the current research, while the results are contradictory to the propositions of the EPPM. The EPPM considers fear as an essential element for connecting cognitive factors and recommended protective behavior and thus sees fear’s potential to explain why some people adopt or ignore health-protective behaviors in the face of risk-related information. However, the results of this research indicated that fear did not have a meaningful mediation effect despite the statistically significant indirect effect. The context of this research may be one possible explanation for such inconsistency: the EPPM has been applied in numerous health studies which covered a wide range of topics such as using condom to prevent HIV/AIDS, smoking, asthma intervention, breast cancer self-examination, and H1N1 influenza. The majority of the diseases examined in previous research have some common characteristics, which are as follows: (a) they are non-emergent public health issues, (b) people are not strangers to these diseases, especially those that are frequently seen in various health campaigns, and (c) modern medicine has done much in the fields of these diseases, and there are established treatments. Although H1N1 influenza does not have the aforementioned characteristics, it did not spread globally in such a fast and devastating way as COVID-19 did. In the early stage of the pandemic when little is known about it, fear may not be the only dominating emotion, as the uncertainty about and rapid spread of the pandemic also induced a high level of negative emotions other than fear, such as worry, anxiety, sadness, anger, stress, and depression (Aslam et al., 2020; Kleinberg et al., 2020; Zhang et al., 2020). Therefore, these emotions could be alternative factors mediating the relationship between risk perceptions and protective behaviors. In fact, in their systematic review of studies on fear appeals, Witte and Allen (2000) have indicated how other affective factors such as irritation, disgust, and puzzlement can influence people’s health-protective behaviors. In addition, drawing on the cognitive appraisal theory of emotion and dispositional coping style, So (2013) have proposed a revised EPPM, namely, E-EPPM, incorporating the construct of anxiety into the original model as a mediator and the concept of monitoring and blunting coping style as a moderator. Besides this, a significant number of fear appeal studies were conducted “in a laboratory setting where exposure to fear appeal is forced on participants” (Witte, 1996a), while the natural setting, which is more complex, increased the likelihood of contradictory results. The COVID-19 pandemic not only posed a challenge for public health but also provided a chance for researchers to reexamine the EPPM in a natural context for improvement.

Media and personal networks are two major sources of risk-related information (Frewer et al., 2002), and the information from these two sources has distinct characteristics. News media presents official information regarding the risks, whereas a personal network offers unfiltered and sometimes unique information from grassroots that may not be available in other channels (Sutton et al., 2008). By incorporating both of these amplification stations into the framework of the EPPM, this study made an attempt to understand the comprehensive picture of the cognitive mechanism underlying the effects of information exposure on people’s health-protective behaviors in the context of a worldwide infectious disease, which was called for in previous studies (e.g., Zhang et al., 2015; Yoo et al., 2016).

## Conclusion

This research highlighted the role of personal network as an important amplification station and revealed its impact on risk-related perceptions. Although the media is a common amplification station of risk information, the current research suggests that the personal network also serves as an amplification station that can influence subsequent perceptions and protective behaviors in people. This study also investigated how perceived efficacy and threat associated with fear, and the results indicated that a higher level was related to less fear, while more perceived threat was associated with more fear. Additionally, the mediating role of fear was tested, and the results did not support a meaningful mediation effect, which contradicted the EPPM. This research provided the following implications: First, individuals are suggested to obtain sufficient and reliable information so as to accurately evaluate the threats of the risks, the effectiveness of solutions as well as their abilities of successfully practicing these solutions. The comprehensive understanding about this information helps in reducing the uncertainties regarding the pandemic and avoiding excessive fear. Second, policymakers are expected to pay more attention on education on media literacy and enhancement of media’s transparency. Since the outbreak of COVID-19, the threats came not only from the pandemic but also from the “infodemic”—“the rapid and far-reaching spread of information of questionable quality” (Gallotti et al., 2020, p. 1285). The selective reporting and misinformation from various media heavily influenced people’s judgment regarding the pandemic and subsequent coping behaviors. If the media is not regarded as trustworthy, the public will turn to other channels (e.g., personal network) which may be filled with unverified information. Therefore, the government should put more emphasis on improving the media literacy of the public, especially those who are more vulnerable to the infodemics (e.g., the elderly); the ability of critically evaluating the messages from different sources is crucial to protect individuals from the infodemics and for them to maintain their mental health amidst the pandemic. Third, this study also offered implications for practitioners. The negative relationship between fear and protective behaviors highlighted the careful use of fear appeal in health campaigns as overuse of fear can backfire and influence the effectiveness of the campaign. In addition, the spread of COVID-19 news on various media has been dominated by negatively framed information (Olagoke et al., 2020), especially in the early stage of the pandemic, such as uncertainties concerning the virus–host interaction and increased cases and fatalities. Given the results that increasing perceived efficacy is helpful for reducing the fear and engaging in protective behaviors, it is suggested that media practitioners provide balanced information so as to enhance the confidence of the public. Lastly, this research has some theoretical implications. Although fear is a core concept in the EPPM, it is “typically treated as manipulation checks rather than independent variables or mediators” (Tannenbaum et al., 2015, p. 3). The examination on the mediation effect of fear revealed the potential for the improvement of the EPPM. Moreover, SAT is regarded as “not a testable theory” (Raupp, 2014, p. 567) but a template to “integrate partial theories and research” (Pidgeon and Henwood, 2010, p. 57), whereas the EPPM is a useful theory to “guide decisions almost every step of communication campaign’s design, implementation, and evaluation” (Popova, 2012, p. 469). This research integrated SAT and EPPM and provided an initial attempt for a new framework that can be used in the context of global pandemic. In summary, this research not only contributed to risk communication studies but also provided useful implications to media and public health practitioners as well as policymakers.

This study has some limitations that should be addressed by future researchers. Its use of cross-sectional data limited the ability to make causal inferences among key variables. Despite the fact that this study’s framework was rooted in robust, mature theories, longitudinal panel data may contribute more solid results and valuable insights. This study measured only people’s health-protective behaviors, but not their maladaptive responses to risk-related information. Future research could more directly and comprehensively test the parameters of the EPPM by measuring people’s maladaptive behaviors. This research did not include any moderators in its model, even though plenty of variables have been found to be significant moderators in the relationships covered in this study—e.g., comparative optimism (Kim and Niederdeppe, 2013) and personal experience (Cho et al., 2013). These moderators should be included in future research in order to provide a more comprehensive and complex model.

## Data Availability Statement

The datasets presented in this article are not readily available. The protocol allows us to share data; however, each request must be reviewed by the IRB of School of Journalism and Communication, Nanjing University beforehand. Requests to access the datasets should be directed to XW, wu_xuan@outlook.

## Ethics Statement

The studies involving human participants were reviewed and approved by the School of Journalism and Communication, Nanjing University. Written informed consent for participation was not required for this study in accordance with the national legislation and the institutional requirements.

## Author Contributions

SZ and XW designed the study. XW collected the data, performed statistical analysis, and drafted the initial manuscript. SZ oversaw the research project, reviewed the manuscript, and contributed to the revised manuscript. Both authors discussed the results and contributed to the final manuscript.

## Conflict of Interest

The authors declare that the research was conducted in the absence of any commercial or financial relationships that could be construed as a potential conflict of interest.
